# The impact of environmental cadmium exposure on type 2 diabetes risk: a protocol for an overview of systematic reviews

**DOI:** 10.1186/s13643-019-1246-7

**Published:** 2019-12-06

**Authors:** Julia Hildebrand, Swarni Thakar, Tonya-Leah Watts, Laura Banfield, Lehana Thabane, Joseph Macri, Stephen Hill, M. Constantine Samaan

**Affiliations:** 1 Department of Pediatrics, McMaster University, Hamilton, Ontario Canada; 2 Division of Pediatric Endocrinology, McMaster Children’s Hospital, Hamilton, Ontario Canada; 3 Health Sciences Library, McMaster University, Hamilton, Ontario Canada; 4 Department of Health Research Methods, Evidence, and Impact, McMaster University, Hamilton, Ontario Canada; 5 Department of Anesthesia, McMaster University, Hamilton, Ontario Canada; 6 Centre for Evaluation of Medicines, St. Joseph’s Healthcare, Hamilton, Ontario Canada; 7 Biostatistics Unit, St. Joseph’s Healthcare Hamilton, Hamilton, Ontario Canada; 8 Hamilton Regional Laboratory Medicine Program, Hamilton, Ontario Canada; 9 Department of Pathology and Molecular Medicine, McMaster University, Hamilton, Ontario Canada; 10 Michael G. DeGroote School of Medicine, McMaster University, Hamilton, Ontario Canada

**Keywords:** Cadmium, Type 2 diabetes mellitus, Obesity, Dyslipidemia, Prediabetes, Hypertension, Non-alcoholic fatty liver disease, Overview of systematic reviews, Meta-analysis, Protocol

## Abstract

**Background:**

Type 2 diabetes mellitus (T2DM) is a worldwide epidemic, and while its etiology is polygenic, the role of environmental contaminant exposure in T2DM pathogenesis is of increasing importance. However, the evidence presented in systematic reviews on the relationship between cadmium exposure and T2DM development is inconsistent. This overview aims to assess existing evidence from systematic reviews linking cadmium exposure to T2DM and select metabolic disorders in humans.

**Methods:**

Searches will be conducted in Medline, Embase, Web of Science, GEOBASE, BIOSIS Previews, and Cochrane Database of Systematic Reviews. Two reviewers (J.H and S.T.) will independently complete screening, data abstraction, risk of bias evaluation, and quality assessment. The primary outcome will be the association between cadmium exposure and T2DM prevalence. Secondary outcomes will include prediabetes, obesity, dyslipidemia, hypertension, and non-alcoholic fatty liver disease. We will perform a meta-analysis if two or more studies assess similar populations, utilize analogous methods, have related study designs, and evaluate similar outcomes.

**Discussion:**

This overview will assess current evidence from systematic reviews for the association between cadmium exposure and risk of T2DM and other metabolic morbidities. This overview may be helpful for policy-makers and healthcare teams aiming to mitigate T2DM risk in populations at risk of cadmium exposure.

**Systematic review registration:**

PROSPERO CRD42019125956

## Background

Over the past few decades, type 2 diabetes mellitus (T2DM) has become a worldwide epidemic [1], with 451 million adults estimated to have T2DM as of 2017 [1]. While obesity is considered the T2DM epidemic’s main driver, T2DM pathogenesis is complex and is not fully understood [2].

One emerging factor implicated in T2DM risk is environmental exposure to endocrine-disrupting chemicals (EDCs) [3–5]. These compounds alter endocrine function by binding to hormone receptors and act as agonist or antagonist signaling molecules or by interfering with hormonal signaling pathways [4, 6–9]. These molecules encompass a vast array of natural and man-made compounds that may exert powerful metabolic effects.

Cadmium (Cd) is a naturally occurring soft metal with endocrine-disrupting properties [6]. It has several industrial uses, including metal mining and the manufacturing of protective steel plating, pigments, and rechargeable batteries. Additional sources of exposure also include phosphate fertilizers, waste disposal, pulp and paper production, and sewage treatment by-products [6, 10]. The adverse effects Cd poses to human, wildlife, and environmental health are significant, and it is classified as a carcinogen by the European Chemicals Agency [11] and as a hazardous substance and air pollutant by the Environmental Protection Agency and in the Clean Water Act and Clean Air Act in the United States[10, 12, 13]. Cd is not involved in normal human physiology, and its adverse health effects range from renal damage, cancer, osteomalacia, and osteoporosis with low-level chronic exposure [10, 14–18] to severe pulmonary damage or even death with acute high-dose exposure [10, 19].

While in-vivo and in-vitro studies suggest a link between Cd exposure and diabetes [20–26], human studies are limited and have yielded conflicting evidence to verify this relationship [27–34].

The purpose of this overview is to synthesize current evidence from systematic reviews for the association between Cd exposure and risk of T2DM and other metabolic outcomes in humans.

## Research questions

### Primary

In the general population, is environmental cadmium exposure associated with increased T2DM risk?

### Secondary

In the general population, is cadmium exposure associated with obesity, prediabetes, dyslipidemia, hypertension, and non-alcoholic fatty liver disease (NAFLD)?

## Methods

This protocol was developed following the Preferred Reporting Items for Systematic Review and Meta-Analysis Protocols (PRISMA-P) statement guidelines [35] (Additional file 1).

### Literature search

The search strategy will be developed by a Health Sciences Librarian with expertise in systematic review search methodologies. To identify systematic reviews relevant to the research question, searches will be conducted in Medline, Embase, Web of Science, BIOSIS Previews, GEOBASE (Geoscience Literature Research Database) [36], and Cochrane Database of Systematic Reviews. A sample Medline search strategy is presented in Table 1. Search strategies will include database-specific terms and filters pertaining to systematic reviews, meta-analyses, cadmium, humans, T2DM, and the metabolic abnormalities listed above. Reference lists of accepted articles and relevant non-systematic reviews will be screened for additional studies.

**Table 1:** MEDLINE search strategy

#	Searches
1	Cadmium/
2	exp cadmium compounds/
3	Cadmium poisoning/
4	cadmium.mp.
5	or/1–4
6	exp diabetes mellitus, type 2/
7	((Type 2 or type II or non-insulin or non-insulin or adult onset or matur* onset) adj3 diabet*).mp.
8	T2DM.mp.
9	T2D.mp.
10	NIDDM.mp.
11	6 or 7 or 8 or 9 or 10
12	Overnutrition/or obesity/or obesity, abdominal/or obesity, morbid/or pediatric obesity/
13	obes*.mp.
14	Overweight/
15	(Overweight or overweight or excess* weight).mp.
16	Overnutrition.mp.
17	(Weight adj2 (gain* or increase* or excess*)).mp.
18	BMI.mp.
19	Body mass index/or skinfold thickness/or waist-hip ratio/
20	Body mass index.mp.
21	Waist circumference*.mp.
22	Waist to hip ratio*.mp.
23	Waist hip ratio*.mp.
24	Waist to height.mp.
25	Waist height.mp.
26	Skinfold thickness/
27	Skinfold*.mp.
28	Insulin resistance/ or metabolic syndrome/
29	(Insulin adj2 (resistan* or sensitiv*)).mp.
30	Metabolic syndrom*.mp.
31	Exp lipid Metabolism/
32	Lipid*.mp.
33	(Lipogenesis or lypolysis or lipoylation).mp.
34	Exp glucose/
35	glucose.mp.
36	Insulin/
37	Hypertension/
38	Hypertens*.mp.
39	or/12–37
40	11 or 39
41	5 and 40
42	Remove duplicates from 41
43	5 and 11
44	Systematic review*.mp.
45	Meta-Analysis as topic/or “review literature as topic”/
46	Meta-analys*.mp.
47	or/44–46
48	43 and 47

We will use Endnote X7 [37] to collect identified articles and de-duplicate them before exporting unique records to an Excel spreadsheet.

### Inclusion criteria

Systematic reviews that synthesize evidence for the association between Cd exposure and diabetes using observational studies with prospective/retrospective cohort, cross-sectional, and case-control study designs will be considered for this review. A systematic review is defined as a review that (1) has a detailed, comprehensive, and systematic search strategy, (2) contains specific inclusion/exclusion criteria and uses systematic review methodology, (3) attempts to synthesize all relevant studies on the topic, and (4) may or may not include a meta-analysis [38].

The population of interest include both males and females in the general population with no restrictions on age, smoking status, ethnicity, geographical location, setting, or timing of publication. The exposure of interest is Cd that is measured in blood or urine samples, and all Cd exposure sources will be considered including smoking, food, water, air, soil, industrial, or accidental exposure. Our primary outcome is T2DM prevalence.

While T2DM accounts for around 90% of diabetes cases in adults globally [1], if a review does not differentiate between type 1 and 2 diabetes, we will reach out to the principal investigators to seek clarification on the exact type of diabetes in the study population in question. Our a priori assumption is that the published data are mainly relevant to T2DM, while acknowledging the limitation if the exact type of diabetes is not reported.

### Exclusion criteria

We will exclude abstracts of systematic reviews with no published full-text papers and non-systematic narrative literature reviews. We will also exclude systematic reviews not published in English, those reporting only on gestational or type 1 diabetes, and non-human or in-vitro studies, if retrieved in the literature searches.

### Study selection

**Fig. 1:**
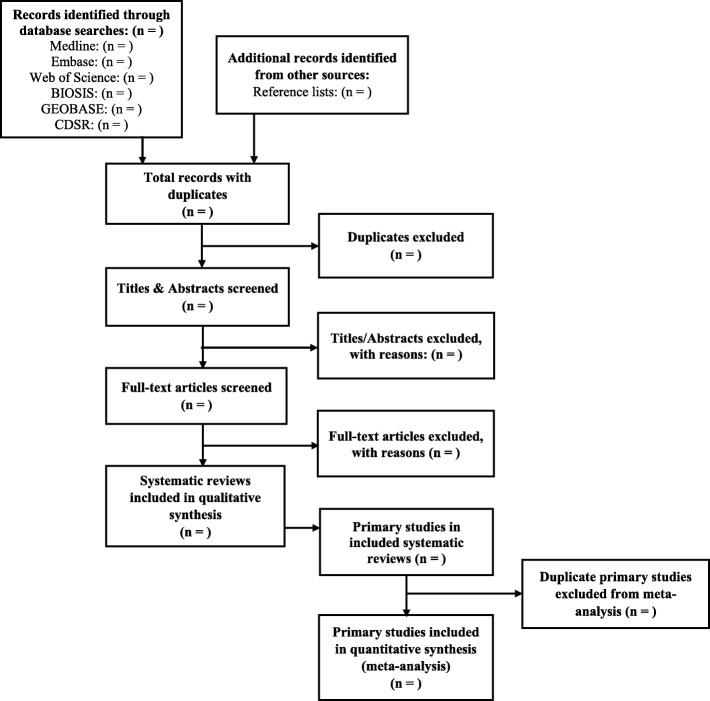
PRISMA Flow Diagram

Two independent reviewers (J.H. and S.T.) will screen titles and abstracts of each record, followed by full-text screening. Reviewers will accept records based on their relevance to the research question and the eligibility criteria. After each stage, the reviewers will discuss discrepancies to reach consensus. To resolve persistent disagreements, a third reviewer will be consulted to make a final decision for study inclusion in the review. The kappa statistic will be calculated for study selection to test interrater reliability. We will use a PRISMA flowchart (Fig. 1) to document the screening process and report this in the full review [39, 40].

Following screening, data abstraction for eligible full-text articles and their included primary studies will be performed. This will be followed by risk of bias (ROB) and quality assessments [41, 42].

### Outcomes

#### Primary

The primary outcome is the association of Cd exposure with T2DM prevalence. We will maintain a broad approach for identifying T2DM to account for varying definitions present within included systematic reviews. T2DM may be defined biochemically using national guidelines of fasting or random plasma glucose, oral glucose tolerance test (OGTT), or HbA1c [43, 44]. In addition, T2DM may be identified through diabetes registries or databases, self- or physician-reported diagnosis, or antidiabetic medication use [45].

#### Secondary

Adverse metabolic effects linked to Cd exposure will be assessed including:

1. Prediabetes, including impaired fasting glucose and impaired glucose tolerance, is defined by fasting or random plasma glucose, OGTT, or HbA1c using national guideline cutoffs [43, 44]. Alternatively, self-reported or physician-reported diagnosis, hospital database diagnosis, or specifically reported prediabetes-related medication or intervention use may be used as a marker of prediabetes.
2. Overweight or obesity is defined using body mass index (BMI) z-score on 85–94.9th percentile indicating overweight and ≥ 95th percentile for obesity in children [46] and BMI 25–29.9 kg/m^2^ indicating overweight and ≥ 30 kg/m^2^ indicating obesity in adults [47].
3. Dyslipidemia is assessed by detecting abnormal levels of total cholesterol, high-density lipoprotein cholesterol, triglycerides, low-density lipoprotein cholesterol (measured or calculated), non-high-density lipoprotein cholesterol, or apolipoprotein A and B measured in, or converted to, mmol/L [48].
4. Hypertension is defined in children as systolic and/or diastolic blood pressure (BP) ≥ 95th percentile according to age- and sex-specific BP centiles and reported in millimeters of mercury (mmHg) [49]. Hypertension will be defined in adults using cutoffs in mmHg from the American College of Cardiology/American Heart Association guidelines [50].
5. NAFLD is defined by elevated transaminases which are threefold higher than the normal reference range of the reported result or documented on liver ultrasound or both [43, 51].

### Data collection

The two independent reviewers (J.H. and S.T.) will collect the data, and kappa will be calculated for interrater reliability. We will develop and pilot a data abstraction form that includes data on authors’ names, journal name, publication date, study setting, country, research question/aims, search strategy details, inclusion and exclusion criteria, and funding agency if applicable. We will also collect data on the population including sample size and participant characteristics including age, sex, ethnicity, BMI, diabetes definition, reports of overweight status, and number of T2DM cases and controls if available, number of included primary studies and their study designs, methods for quality assessment of primary studies, Cd exposure and measurement, and conclusions. We will also extract the pooled effect estimates along with 95% confidence interval (CI) and the *I*^2^ values and chi-square test *p* value if reported for heterogeneity across individual studies.

Furthermore, we will collect data from primary studies in each systematic review including authors’ names, publication year, study design, population characteristics (ethnicity, age, sex), country, diabetes definition, participant inclusion and exclusion criteria, number of participants, assessment method, and data for Cd exposure and for each outcome. The standard mean difference and 95% CI for the desired outcome, if available, will also be collected. If there are missing data, we will e-mail the principal investigators of the systematic reviews to determine if the data are accessible. If the data are not available to them, we will contact the authors of the primary studies from which the evidence was derived in the reported systematic reviews to ascertain if the data are available [52].

### Risk of bias and quality assessment

Two reviewers (J.H and S.T.) will independently assess the methodological quality of systematic reviews using a revised version of the Assessment of Multiple Systematic Reviews-2 (AMSTAR-2) tool [53] (Additional file 2). AMSTAR-2 was designed for systematic reviews of intervention studies [53]. Thus, we have modified it for appropriate quality assessment of systematic reviews of observational studies (Additional file 2) based on a previously modified R-AMSTAR tool [54]. This tool consists of 14 items and is not meant to provide an overall score. Rather, authors will consider the effect of low scores for certain items on review quality and provide a high, moderate, low, or critically low overall confidence rating based on weaknesses in these critical domains [53].

ROB of included systematic reviews will also be assessed using the risk of bias in systematic reviews (ROBIS) tool [55]. This tool measures ROB as high, low, or unclear in four domains (study eligibility criteria, identification and selection of studies, data collection and study appraisal, and synthesis and findings) [55].

### Statistical analysis

To avoid confounding from having the same primary study included in multiple systematic reviews, a meta-analysis will be conducted at the primary study level if applicable. For this process, primary studies of each review will be screened, and all duplicates will be removed. A meta-analysis will be performed if two or more primary studies with similar methods, populations, exposures, and outcomes are identified from included reviews. We will use a random effects model to account for variation in effect size among studies [56]. Dichotomous outcomes will be reported as an odds ratio and continuous outcomes as a standardized mean difference with 95% CI. As the systematic reviews in this overview would have included observational studies, we will perform our analyses based on method of Cd measurement and study designs including cross-sectional, case-control, or cohort studies. A meta-analysis will be performed provided that at least two studies are available under each category.

If a meta-analysis is performed, heterogeneity among included systematic reviews will be quantified using the inconsistency index (*I*^2^) and *p* values from the chi-square test for homogeneity. The *I*^2^ will be interpreted using the Cochrane Collaboration threshold [56], with a value of > 75% representing considerable heterogeneity. A chi-square test *p* value of < 0.10 will define statistical significance [56]. If a meta-analysis is not feasible, we will provide narrative and tabulated summaries of the data.

Several studies have demonstrated sex-specific effects of Cd exposure [57–62], and Cd exposure levels may vary based on geographic location and method of exposure. Therefore, we will perform subgroup analyses by sex and ethnicity (stratified by countries where primary studies were carried out). We will also perform a subgroup analysis for accidental/occupational exposure compared to exposure among the general population if possible and will ask investigators for unpublished data.

If considerable heterogeneity is observed, we will perform meta-regression analyses to investigate the potential bases of heterogeneity if data from ten or more studies are available [56].

If ten or more studies are identified for an outcome a sensitivity analysis will be conducted, and studies with high risk of bias and small sample size will be excluded in a separate meta-analysis to assess their impact on the results using the Review Manager Software version 5.3 (RevMan 5.3) [63]. We will also evaluate publication bias using a funnel plot and employ visual inspection and Egger’s test to determine plot asymmetry using the Statistical Package for the Social Sciences (SPSS) version 25.0 [64, 65].

Results of this overview will be reported according to the Preferred Reporting Items for Systematic Reviews and Meta-Analyses (PRISMA) guidelines [39, 40]. The reasons for any protocol amendments will be documented in the full review.

## Discussion

While Cd exposure may occur through industrial exposures or via consumption of contaminated food and water, and soil or dust contact [66], the primary source of human exposure is smoking, which almost doubles Cd body burden [10]. In non-smokers, the primary Cd exposure source is diet, with the largest concentrations acquired from plant foods [10, 67, 68], liver/kidney meats, and shellfish [10, 14, 66, 69, 70]. In areas near Cd-emitting industries, well water and rivers may also represent significant exposure sources [71].

The role of Cd in T2DM development remains unclear. Multiple mechanisms have been proposed including increased gluconeogenic enzyme activation, increased oxidative stress in pancreatic beta-cells, altered glucose transport in adipose and renal tissues, and altered cell-cell adhesions that can potentially lead to islet dysfunction, impaired insulin secretion, and subsequent dysglycemia [72–74]. As T2DM rates rise worldwide [75], accurately determining Cd’s contribution to diabetes risk could improve decision-making regarding Cd use and precautions to protect at-risk populations.

One limitation of this overview is the exclusion of non-English language articles, which may exclude some additional papers. Nonetheless, this overview will clarify the association between Cd exposure and T2DM in the general population, help identify future research needs, and guide health policy to limit exposure if an association between Cd exposure and T2DM risk is confirmed.

## Supplementary information

**Additional file 1.** PRISMA-P checklist. This checklist documents the location within this protocol of recommended items to address in a systematic review protocol.

**Additional file 2.** Modified AMSTAR-2 tool. This file contains a modified version of the AMSTAR-2 tool, as well as a list of the modifications made by the authors to the original tool.

## Data Availability

Not applicable.

## Abbreviations

AMSTAR-2: Assessment of Multiple Systematic Reviews 2
BMI: Body mass index
BP: Blood pressure
Cd: Cadmium
CI: Confidence intervals
EDC: Endocrine-disrupting chemical
HbA1c: Hemoglobin A1c
NAFLD: Non-alcoholic fatty liver disease
OGTT: Oral glucose tolerance test
PRISMA: Preferred Reporting Items for Systematic Reviews and Meta-Analyses
PRISMA-P: Preferred Reporting Items for Systematic Review and Meta-Analysis Protocols
RevMan 5.3: Review Manager Software version 5.3
ROB: Risk of bias
ROBIS: Risk of bias in systematic reviews
SPSS: Statistical Package for the Social Sciences
T2DM: Type 2 diabetes mellitus
