# Supplementary material for: The impact of environmental cadmium exposure on type 2 diabetes risk: a protocol for an overview of systematic reviews
Source: Syst Rev. 2019 Dec 6;8:309. doi: 10.1186/s13643-019-1246-7 (PMC6896588; doi:10.1186/s13643-019-1246-7)
Supplement: Supplementary file 2 — Additional file 2. Modified AMSTAR-2 tool. This file contains a modified version of the AMSTAR-2 tool, as well as a list of the modifications made by the authors to the original tool. [file 13643_2019_1246_MOESM2_ESM.pdf]

Modified AMSTAR 2: a tool that has been modified from the original AMSTAR 2 to be appropriate for assessing the methodological quality of systematic reviews that include observational study designs.

1. **Did the research questions and inclusion criteria for the review include the components of PICO?**

For Yes:
- ☐ Population
- ☐ Intervention/Exposure
- ☐ Comparator group (if relevant)
- ☐ Outcome

Optional (may not be relevant)
- ☐ Timeframe for follow-up

- ☐ Yes
- ☐ No

---

2. **Did the report of the review contain an explicit statement that the review methods were established prior to the conduct of the review and did the report justify any significant deviations from the protocol?**

For Partial Yes:
The authors state that they had a Written Protocol or guide that included ALL the Following:
- ☐ Review question(s)
- ☐ A search strategy
- ☐ Inclusion/exclusion criteria
- ☐ A risk of bias assessment

For Yes:
As for partial yes, plus the protocol should be registered and should also have specified:
- ☐ a meta-analysis/synthesis plan, if appropriate, *and*
- ☐ a plan for investigating causes of heterogeneity
- ☐ Justification for any deviations from the protocol

- ☐ Yes
- ☐ Partial Yes
- ☐ No

---

3. **Did the review authors use a comprehensive literature search strategy?**

For Partial Yes (all of the following):
- ☐ Searched at least 2 databases (relevant to research question)
- ☐ Provided key word and/or Search strategy
- ☐ Justified publication restrictions (e.g. language)

For Yes, should also have (all the following):
- ☐ Searched the reference lists/bibliographies of included studies
- ☐ Searched trial/study registries
- ☐ Included/consulted content experts in the field
- ☐ Where relevant, searched for grey Literature
- ☐ Conducted search within 24 months of Completion of the review

- ☐ Yes
- ☐ Partial Yes
- ☐ No

---

4. **Did the review authors perform study selection in duplicate?**

For Yes, either ONE of the following:
- ☐ At least two reviewers independently agreed on selection of eligible studies and achieved consensus on which studies to include
- ☐ OR two reviewers selected a sample of eligible studies <u>and</u> achieved good agreement (at least 80 percent), with the remainder selected by one reviewer.

- ☐ Yes
- ☐ No

---

5. **Did the review authors perform data extraction in duplicate?**

For Yes, either ONE of the following:
- ☐ At least two reviewers achieved consensus on which data to extract from included studies
- ☐ OR two reviewers extracted data from a sample of eligible studies <u>and</u> achieved good agreement (at least 80 percent), with the remainder extracted by one reviewer

- ☐ Yes
- ☐ No

Modified AMSTAR 2: a tool that has been modified from the original AMSTAR 2 to be appropriate for assessing the methodological quality of systematic reviews that include observational study designs.

---

6. Did the review authors provide a list of excluded studies and justify the exclusions?

For Partial Yes:	For Yes, must also have:	
☐ Provided a list of all potentially relevant studies that were read In full-text form but excluded From the review	☐ Justified the exclusion from the review of each potentially relevant study	☐ Yes<br>☐ Partial Yes<br>☐ No

---

7. Did the review authors describe the included studies in adequate detail?

For Partial Yes (ALL the following):	For Yes, should also have ALL the following:	
☐ Described populations	☐ Described population in detail	☐ Yes
☐ Described exposures	☐ Described exposure in detail	☐ Partial Yes
☐ Described comparators (if present)	☐ Described comparator in detail	☐ No
☐ Described outcomes	☐ Described study's setting	
☐ Described research designs	☐ Timeframe for follow-up (if applicable)	

---

8. Did the review authors use a satisfactory technique for assessing the scientific quality and risk of bias (ROB) in individual studies that were included in the review?

For Partial Yes, must have assessed ROB:	For Yes, must have also assessed ROB from:	
☐ From confounding, *and*<br>☐ From selection bias<br>☐ From information bias<br>☐ From measurement bias	☐ methods used to ascertain exposures and outcomes, *and*<br>☐ selection of the reported result from among multiple measurements of analyses of a specified outcome	☐ Yes<br>☐ Partial Yes<br>☐ No

---

9. Did the review authors report on the sources of funding for the studies included in the review?

For Yes	
☐ Must have reported on the sources of funding for individual studies included in the Review. Note: Reporting that the reviewers looked for this information but it was not reported by study authors also qualifies	☐ Yes<br>☐ No

---

10. If meta-analysis was performed did the review authors use appropriate methods for statistical combination of results?

For Yes:	
☐ The authors justified combining the data in a meta-analysis<br>☐ AND they used an appropriate weighted technique (ie. random effects model) to combine study results, adjusting for heterogeneity if present<br>☐ AND they statistically combined effect estimates from studies that were adjusted for confounding, rather than combining raw data, or justified combining raw data when adjusted effect estimates were not available	☐ Yes<br>☐ No<br>☐ No meta-analysis conducted

---

11. If meta-analysis was performed, did the review authors assess the potential impact of ROB in individual studies on the results of the meta-analysis or other evidence synthesis?

For Yes:	
☐ If the pooled estimate was based on studies at variable ROB, the authors performed analyses to investigate possible impact of ROB on summary estimates of effect	☐ Yes<br>☐ No<br>☐ No meta-analysis conducted

12. Did the review authors account for ROB in individual studies when interpreting/discussing the results of the review?

For Yes:

☐ The review provided a discussion of the likely impact of ROB on the results	☐ Yes<br>☐ No

---

13. Did the review authors provide a satisfactory explanation for, and discussion of, any heterogeneity observed in the results of the review?

For Yes:

☐ There was no significant heterogeneity in the results	☐ Yes
☐ OR if heterogeneity was present the authors performed an investigation of sources of any heterogeneity in the results and discussed the impact of this on the results of the review	☐ No

---

14. If they performed quantitative synthesis did the review authors carry out an adequate investigation of publication bias (small study bias) and discuss its likely impact on the results of the review?

For Yes:

☐ Performed graphical or statistical tests for publication bias and discussed the likelihood and magnitude of impact of publication bias	☐ Yes<br>☐ No<br>☐ No meta-analysis conducted

---

Citation of original AMSTAR-2 tool: Shea BJ, Reeves BC, Wells G, Thuku M, Hamel C, Moran J, Moher D, Tugwell P, Welch V, Kristjansson E, Henry DA. AMSTAR 2: a critical appraisal tool for systematic reviews that include randomised or non-randomised studies of healthcare interventions, or both. BMJ. 2017 Sep 21;358:j4008.

Modifications made to original AMSTAR-2 tool:

For the purposes of this overview, several items of the AMSTAR-2 tool were modified:

- Firstly, item 3 was removed as it is only relevant for systematic reviews that include randomized control trials (RCTs).
- Secondly, items 1 and 8 were modified to include 'exposure' along with intervention in PICO criteria, to make the criteria appropriate for observational studies that assess exposures.
- Furthermore, item 9 was adjusted to make risk of bias considerations specific to observational studies (selection bias, information bias, measurement errors, confounding).
- As well, criteria specific to RCTs was removed from items 11, 12, and 13.
- Finally, item 16 was removed as conflicting interests are rarely reported in observational studies.
